# Single Radial Immunodiffusion (SRID) Assay for Quantitative Determination of Recombinant SOD Protein in a Brucellosis Vaccine Candidate: Method Development and Validation

**DOI:** 10.3390/mps9040113

**Published:** 2026-07-28

**Authors:** Gulnur Nakhanova, Olga Chervyakova, Kamshat Shorayeva, Aigerim Zhakypbek, Sabina Moldagulova, Aknur Ulankyzy, Alisher Omurtay, Yeraly Shayakhmetov, Temirlan Baiseit, Zharkinay Absatova, Aisha Issabek, Gaukhar Shynybekova, Sandugash Sadikaliyeva, Aziz Nakhanov, Kuanysh Jekebekov, Ainar Kossylganova, Karlygash Zhaparkulova, Assem Kalykova, Tolkyn Bekezhanova, Albina Atakanova, Zakir Yershebulov, Kuandyk Zhugunissov

**Affiliations:** 1 Research Institute for Biological Safety Problems, National Holding QazBioPharm, Gvardeiskiy 080409, Kazakhstan; g.nakhanova@biosafety.kz (G.N.); o.chervyakova@biosafety.kz (O.C.); k.shorayeva@biosafety.kz (K.S.); a.zhakypbek@biosafety.kz (A.Z.); a.ulankyzy@biosafety.kz (A.U.); a.omurtay@biosafety.kz (A.O.); e.shayahmetov@biosafety.kz (Y.S.); t.baiseit@biosafety.kz (T.B.); zh.absatova@biosafety.kz (Z.A.); a.issabek@biosafety.kz (A.I.); sh.gaukhar@biosafety.kz (G.S.); s.sadikalieva@biosafety.kz (S.S.); a.nakhanov@biosafety.kz (A.N.); k.zhekebekov@biosafety.kz (K.J.); k.zhugunisov@biosafety.kz (K.Z.); 2 School of Pharmacy, Asfendiyarov Kazakh National Medical University, Almaty 050000, Kazakhstan; kossylganova.a@kaznmu.kz (A.K.); zhaparkulova.k@kaznmu.kz (K.Z.); a.kalykova@gmail.com (A.K.); bekezhanova.t@kaznmu.kz (T.B.); atakanova.a@kaznmu.kz (A.A.); 3 Limited Liability Partnership “OtarBioPharm”, QazBioPharm National Holding JSC, Gvardeiskiy 080409, Kazakhstan; ershebulov@mail.ru

**Keywords:** brucellosis, recombinant proteins, single radial immunodiffusion, method validation, vaccine quality control

## Abstract

Recombinant superoxide dismutase (SOD) protein is a promising antigen candidate for brucellosis vaccines, and its quantitative determination is essential for vaccine quality control, standardization, and regulatory compliance. Although enzyme-linked immunosorbent assay (ELISA) is widely used for antigen quantification, its application to vaccine formulations may be limited due to chemical modifications of antigenic determinants occurring during detoxification and adjuvantation processes. Therefore, alternative analytical methods, such as single radial immunodiffusion (SRID), which enable direct antigen quantification independent of antigenic determinant modifications, represent valuable tools for vaccine analysis. The aim of this study was to develop and validate a single radial immunodiffusion (SRID) assay for the quantitative determination of recombinant SOD protein and to obtain hyperimmune sera in sheep. Sheep were immunized with recombinant SOD protein formulated with aluminum hydroxide or AddaS03 adjuvants. Antibody responses were evaluated using agar gel immunodiffusion (AGID) and enzyme-linked immunosorbent assay (ELISA). Immunization with aluminum hydroxide induced higher antibody titers than AddaS03. By day 42, ELISA titers reached 1:25,600 in the aluminum hydroxide group compared with 1:3200 in the AddaS03 group. The obtained hyperimmune sera were used for SRID assay development. The optimized SRID assay demonstrated high reproducibility and specificity, with no cross-reactivity against unrelated proteins. A linear relationship was observed between the square of the precipitation ring diameter (D^2^) and antigen concentration (R^2^ = 0.956–0.989). The assay showed a limit of detection of 0.312 μg/mL, a linear range of 0.625–10 μg/mL, high precision (CV < 2%), and robustness under varying analytical conditions. The developed SRID assay represents a specific, reproducible, and robust tool for quantitative control of recombinant SOD protein in vaccine formulations.

## 1. Introduction

Brucellosis is a widespread zoonotic infectious disease caused by bacteria of the genus *Brucella*, affecting both animals and humans globally. The disease is characterized by a chronic course, multisystem involvement, and substantial economic losses in livestock production [1]. The principal causative agents of brucellosis in farm animals include *Brucella melitensis*, *Brucella abortus*, and *Brucella suis* [2,3]. Human infection primarily occurs through direct contact with infected animals or consumption of contaminated animal-derived products, particularly in occupational settings and endemic regions [1,2].

According to recent epidemiological data, hundreds of thousands of human brucellosis cases are reported annually worldwide; however, the actual incidence is likely considerably higher due to underdiagnosis and underreporting in endemic regions [3,4,5]. Despite ongoing preventive measures, brucellosis remains a significant public health and veterinary concern in many parts of the world, particularly in countries with extensive livestock farming systems [5,6]. The disease continues to pose a serious challenge in endemic regions, including Kazakhstan, where livestock-associated exposures remain prevalent [6,7].

Vaccination remains the most effective strategy for brucellosis prevention and control. Currently, live attenuated vaccines, such as *Brucella melitensis* Rev.1 and *Brucella abortus* S19 strains, are widely used for prevention in animals. However, these vaccines are not approved for human use due to several significant limitations: (i) residual virulence of vaccine strains, which can cause adverse reactions and chronic infections in immunocompromised individuals; (ii) potential interference with serological diagnostic assays, complicating post-vaccination serological monitoring [8]; and (iii) concerns regarding potential reversion to virulence. These limitations underscore the critical need for safe and effective brucellosis vaccines suitable for human immunization [9].

In recent years, considerable attention has been directed toward recombinant subunit vaccines containing individual antigenic proteins of *Brucella* spp., as these represent a promising alternative to live attenuated vaccines [10]. Recombinant subunit vaccines offer several significant advantages: improved safety profiles through elimination of infectious agents, better tolerability profiles, precise control over antigen composition and dose, reduced or eliminated interference with serological diagnostics (enabling development of DIVA-differentiating infected from vaccinated animals-assays), and potential for multicomponent formulations targeting multiple protective epitopes [8].

Among the numerous *Brucella* antigens under investigation as vaccine candidates, recombinant superoxide dismutase (SOD; Cu/Zn-SOD or BruA) has emerged as a particularly promising target. Superoxide dismutase is an essential bacterial enzyme that catalyzes the dismutation of reactive oxygen species, protecting the pathogen against host-generated oxidative stress during intracellular survival [11]. Beyond its role in bacterial pathogenesis, SOD possesses strong immunogenic properties and has been demonstrated to induce robust humoral and cellular immune responses in vaccination studies [12]. Previous immunological studies have shown that SOD-based immunization provides partial protection against *Brucella* infection in experimental animal models [10], making it an attractive component for multicomponent vaccine formulations.

The development and standardization of recombinant protein-based vaccines require rigorous analytical methods for accurate, precise, and reproducible quantitative determination of antigen content [13,14].

Multiple analytical approaches have been developed for quantitative protein determination and vaccine antigen quantification, each with distinct advantages and limitations.

ELISA-based approaches remain the gold standard for antigen quantification in many vaccine development pipelines [15]. However, ELISA has significant limitations when applied to vaccine formulations. Chemical modifications of antigenic determinants occurring during vaccine processing—particularly during detoxification procedures (e.g., with formaldehyde or glutaraldehyde) and adjuvantation—can substantially alter or mask epitopes recognized by detection antibodies [16]. Consequently, ELISA may underestimate or misrepresent actual antigen content in vaccine formulations containing modified antigens [15].

Immunodiffusion-based methods, particularly single radial immunodiffusion (SRID) and radial immunodiffusion (RID), have been historically employed for antigen quantification and represent reference methods for quality control of several licensed vaccines, including influenza and pertussis vaccines [14,15]. These methods quantify the total antigen content based on the formation of precipitation complexes, making them largely independent of minor modifications to individual antigenic epitopes [14,17]. SRID has demonstrated high reproducibility, simplicity, and ability to quantitatively assess antigen concentration based on the mathematical relationship between precipitation ring diameter and antigen concentration [14,15,17].

Several emerging analytical technologies have recently been explored as alternatives to conventional immunoassays for protein detection and quantification.

Aggregation-induced emission (AIE) is an emerging optical detection strategy in which molecular aggregation enhances fluorescence emission. AIE-based competitive immunoassays have shown promising performance for protein detection owing to their high sensitivity, low background fluorescence, and potential for miniaturization and point-of-care applications [18]. Nevertheless, their application in vaccine quality control remains limited because these methods have not yet been sufficiently validated or standardized for routine analytical use.

In addition to fluorescence-based detection, gold nanoparticle (AuNP)-based colorimetric immunoassays have also attracted considerable interest because of the unique optical properties of AuNPs. These assays provide high analytical sensitivity, rapid visual detection, and the possibility of point-of-care testing without complex instrumentation [19]. However, their analytical performance may be influenced by nanoparticle aggregation, batch-to-batch variability, and the limited validation of these methods for pharmaceutical quality control.

Beyond optical approaches, electrochemical immunosensors have emerged as another promising strategy for antigen detection. Biorecognition element-free electrochemical systems and competitive electrochemical immunosensors enable real-time monitoring of antigen–antibody interactions through electrical signal measurement [20]. These platforms offer high sensitivity, rapid analysis, and opportunities for miniaturization and multiplex detection. However, their routine application remains limited by the need for specialized instrumentation and the potential influence of electroactive compounds present in complex vaccine formulations.

Surface plasmon resonance (SPR) biosensors represent another label-free approach for monitoring biomolecular interactions in real time. SPR-based immunoassays provide high sensitivity and allow the determination of binding kinetics without the use of labeled reagents [21]. However, SPR systems require expensive instrumentation, specialized expertise, and are therefore not routinely used for vaccine quality control.

Despite these advantages, the routine use of these technologies in pharmaceutical quality control remains limited because most have not been sufficiently validated for vaccine antigen quantification, are not included in pharmacopeial guidelines, require specialized equipment and trained personnel, and still present challenges related to interlaboratory standardization and long-term stability of reference materials and reagents.

Although SRID has been in use since the 1960s, it remains highly relevant for vaccine quality control because it directly measures total antigen content without dependence on the preservation of individual epitopes, demonstrates high reproducibility and precision under properly standardized conditions [17], requires minimal instrumentation and technical expertise, facilitating implementation in diverse laboratory settings, particularly in resource-limited regions [14,15], and is recognized by international regulatory authorities, including the European Pharmacopoeia and the United States Pharmacopeia, as an acceptable reference method for vaccine potency determination [22].

However, for each recombinant protein and pharmaceutical formulation, the analytical suitability of the method requires rigorous experimental validation. Although SRID has been previously applied to viral vaccine antigens, data regarding SRID validation for recombinant bacterial proteins—particularly SOD—remain limited [17]. Furthermore, the generation of suitable monospecific hyperimmune antisera, which is critical for assay performance, requires proper immunization protocol development and characterization.

In the present study, we developed, optimized, and comprehensively validated a single radial immunodiffusion assay employing monospecific antiserum for the quantitative determination of recombinant SOD protein in a brucellosis vaccine candidate formulation. Additionally, we describe the generation and characterization of high-affinity hyperimmune sera in sheep through systematic immunization with recombinant SOD protein formulated with different adjuvants. The resulting validated SRID assay provides a reliable, reproducible, and robust analytical tool applicable to routine vaccine quality control and regulatory compliance procedures.

## 2. Materials and Methods

### 2.1. Materials

The study quantified recombinant superoxide dismutase (SOD) protein produced in an Escherichia coli expression system. The recombinant SOD protein was expressed using the pET-28a expression vector and contained an N-terminal hexahistidine (6×His, His_6_) affinity tag to facilitate purification by immobilized metal affinity chromatography (IMAC). The purified recombinant SOD protein was formulated with either aluminum hydroxide or the oil-in-water nano-emulsion adjuvant AddaS03™ (Cat. No. vac-as03-10, InvivoGen, San Diego, CA, USA) for immunization studies.

Recombinant human superoxide dismutase 1 (SOD1) (Recombinant Human SOD1 Protein, Cat. No. NBP2-34940, Novus Biologicals, Centennial, CO, USA) was included as a positive control. The protein was expressed in an *Escherichia coli* expression system and purified to >95% purity according to SDS-PAGE analysis. It is a nonglycosylated polypeptide with an approximate molecular weight of 18.1 kDa. 

Recombinant *Brucella* proteins OMP16, OMP19, L7/L12, and IalB, produced in an Escherichia coli expression system and purified at the Research Institute for Biological Safety Problems, were used as heterologous control antigens to assess cross-reactivity. Bovine serum albumin (BSA) and phosphate-buffered saline (PBS, pH 7.2–7.4) served as negative controls.

### 2.2. Ethical Statement

All experiments involving laboratory animals were conducted in accordance with international and national regulatory requirements, including Directive 2010/63/EU governing the protection of animals used for scientific purposes [23]. The study protocol was approved by the Bioethics Committee of the Research Institute for Biological Safety Problems (Protocol No. 1, dated 24 May 2024).

Animals were housed under standard laboratory husbandry conditions with free access to food and water and were monitored daily throughout the study. Environmental conditions were maintained according to institutional animal care guidelines.

### 2.3. Production of Hyperimmune Sera

Hyperimmune sera against *Brucella* spp. were produced using clinically healthy, brucellosis-seronegative sheep aged 6–12 months and weighing at least 20 kg. Animals were considered healthy based on veterinary clinical examination, including the absence of clinical signs of disease and normal physiological condition, prior to immunization. Immunization was performed using purified *Brucella* spp. antigen previously characterized in terms of specificity and immunoreactivity.

**Figure 1:**
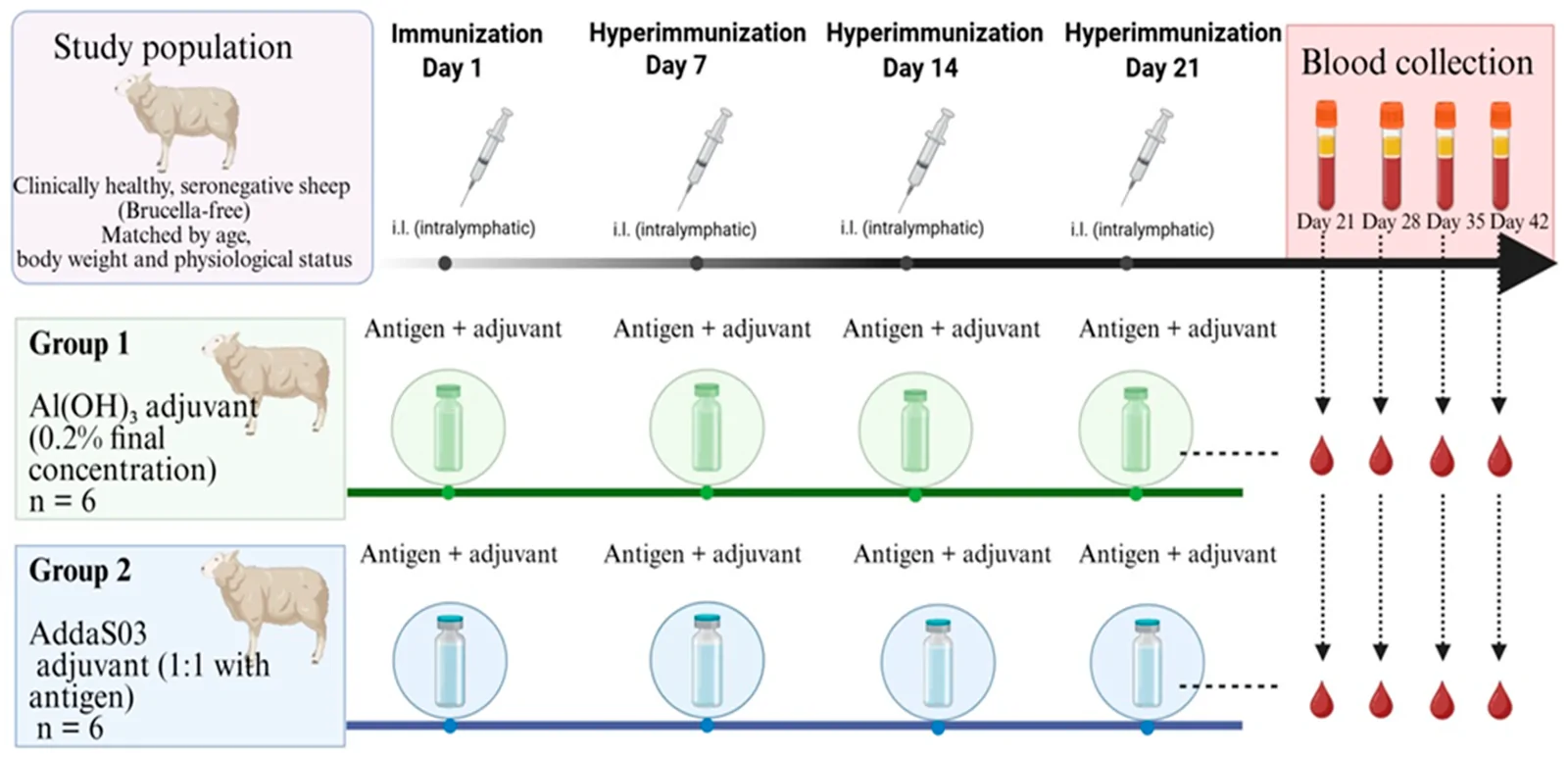
Experimental design of intralymphatic immunization of sheep and blood sampling schedule for the production of hyperimmune sera against recombinant SOD protein. Immunization was performed by intralymphatic (i.l.) administration into the prescapular lymph nodes using a prime–boost regimen consisting of primary immunization (Day 1) followed by booster injections on Days 21, 28, and 35. Each sheep received 100 μg of recombinant SOD protein at each immunization. Blood samples were collected at the corresponding time points to evaluate the dynamics of the humoral immune response. Two experimental groups were established: (i) antigen adsorbed onto aluminum hydroxide (0.2%) and (ii) antigen formulated with the AddaS03 adjuvant system (1:1, *v*/*v*).

Aluminum hydroxide (final concentration 0.2%) and the oil-in-water nano-emulsion adjuvant AddaS03, mixed with the antigen at a 1:1 (*v*/*v*) ratio, were used as adjuvants to compare the effects of different adjuvant platforms on the induction of humoral immune responses according to the immunization scheme presented in Figure 1.

Hyperimmunization was performed according to a prime–boost regimen in which 100 μg of recombinant SOD protein was administered at each immunization. All procedures were carried out in compliance with aseptic techniques and veterinary supervision requirements. Blood samples were collected on Days 21, 28, 35, and 42 after the initiation of immunization. Serum samples were obtained using a standard protocol and stored under regulated conditions until further analysis.

### 2.4. Analysis of Hyperimmune Sera

Agar gel immunodiffusion (AGID) was performed at 24 °C using 1% agarose (BD Difco™, Becton, Dickinson and Company, Franklin Lakes, NJ, USA) prepared in physiological saline supplemented with rivanol. Purified *Brucella* spp. antigen (Brucellosis antigen for CFT Innovative Diagnostics, IDvet, Grabels, France) with an AGID activity titer of 1:8 was used as the test antigen. The serum samples were subjected to twofold serial dilutions ranging from 1:2 to 1:32. Incubation was carried out in a humid chamber, and the results were evaluated after 24–36 h. A positive result was defined by the formation of distinct precipitation lines between antigen and serum wells, whereas no precipitation lines were observed in negative samples [15].

Enzyme-linked immunosorbent assay (ELISA) was performed using the commercial *Brucella* IgG ELISA Kit (MyBioSource, San Diego, CA, USA) for the quantitative determination of *Brucella* spp.-specific immunoglobulin G (IgG) antibodies in serum samples according to the manufacturer’s instructions. Optical density was measured at 450 nm using a microplate spectrophotometer.

### 2.5. Single Radial Immunodiffusion Assay

Single radial immunodiffusion (SRID) was performed in agarose gel according to a modified Mancini method [16]. The gel consisted of 1.5% agarose dissolved in 0.01 M phosphate-buffered saline (PBS, pH 7.2–7.4) containing monospecific antiserum at a concentration of 15 μL/mL.

The standard antigen and test samples were pretreated with a 10% solution of Zwittergent 3-14 detergent at a ratio of 1:10 and incubated at room temperature for 30 min, followed by serial dilution in PBS. A volume of 25 μL of standard and test samples was added to each well. The plates were incubated in a humid chamber at room temperature for 24 h until precipitation rings were formed.

**Figure 2:**
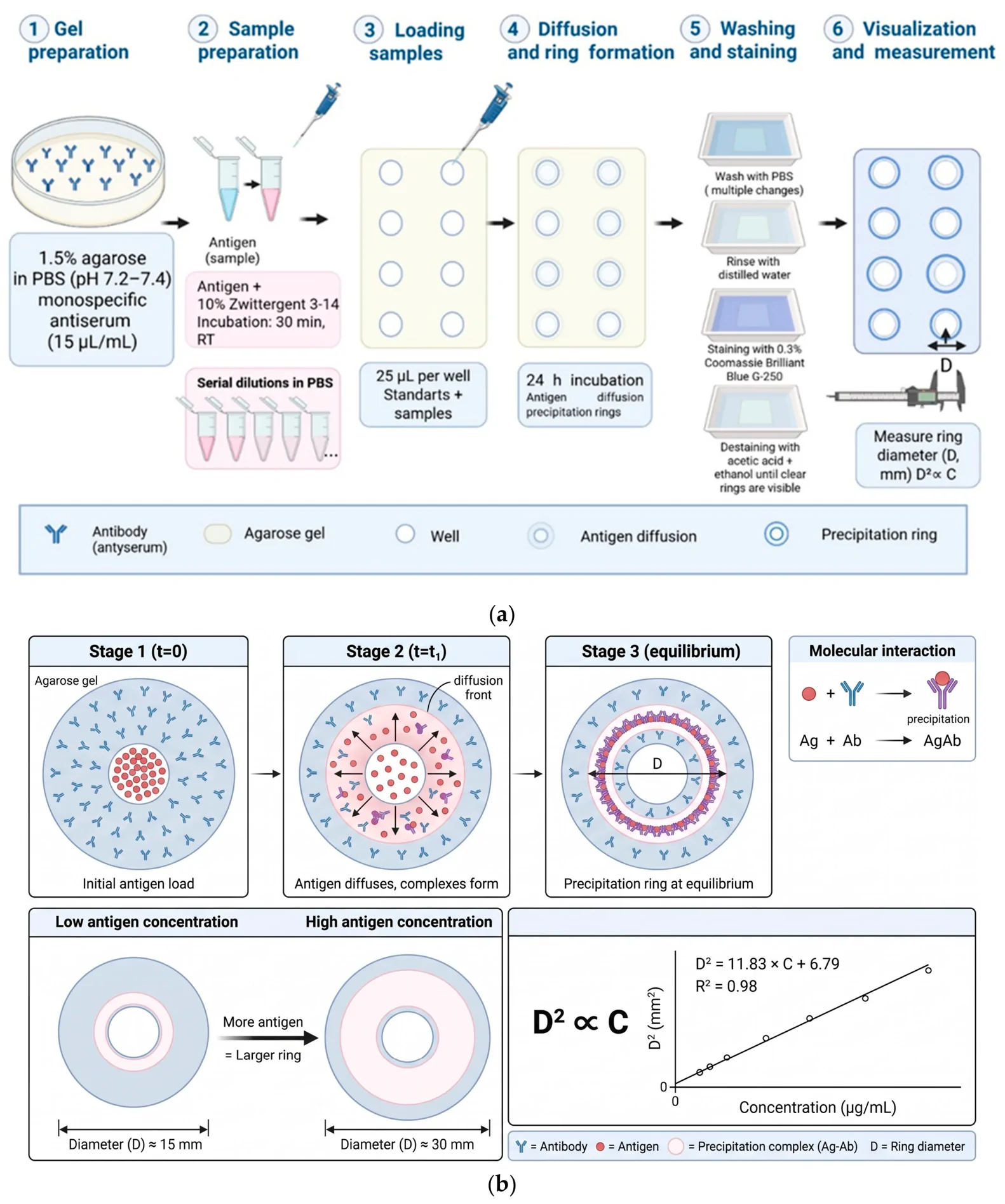
Principle and workflow of the single radial immunodiffusion (SRID) assay. (**a**) Schematic workflow of the SRID assay, including agarose gel preparation with incorporated antiserum, antigen sample preparation and serial dilution, sample loading into the wells, radial antigen diffusion, precipitation ring formation, gel washing, Coomassie Brilliant Blue G-250 staining, and measurement of the precipitation ring diameter (D). (**b**) Schematic representation of the molecular principle of the SRID assay. Following antigen loading, the antigen diffuses radially through the antibody-containing agarose gel and forms insoluble antigen–antibody complexes at the zone of equivalence, resulting in a precipitation ring. The arrows indicate the precipitation ring formed at the zone of antigen–antibody equivalence. Higher antigen concentrations produce larger precipitation rings. Quantitative determination is based on the Mancini principle, according to which the square of the precipitation ring diameter (D^2^) is directly proportional to the antigen concentration (D^2^ ∝ C) [24].

Following the 24 h incubation, the gels were washed five times in phosphate-buffered saline (PBS) for 30–40 min per wash on an orbital shaker at 40–50 rpm, with buffer replacement after each washing step. The gels were subsequently washed twice in distilled water for 15 min each under the same shaking conditions. During all washing steps, a sufficient volume of washing solution was used to ensure that the agarose gels remained completely submerged throughout the procedure. The gels were then stained with 0.3% Coomassie Brilliant Blue G-250, followed by destaining with a mixture of acetic acid and ethanol until clearly distinguishable precipitation zones were obtained (Figure 2).

Quantitative analysis was performed by measuring the diameter of precipitation rings. The square of the ring diameter (D^2^) was considered proportional to antigen concentration, and quantitative determination was carried out using a calibration curve constructed with the reference standard.

### 2.6. Validation of the Single Radial Immunodiffusion (SRID) Assay

#### 2.6.1. Specificity

The specificity of the assay was evaluated based on the ability of antibodies immobilized within the agarose gel to form precipitation rings exclusively in the presence of the target recombinant human superoxide dismutase protein (SOD1) (Recombinant Human SOD1 Protein, NBP2-34940, Novus Biologicals, USA). Working dilutions of the protein (20, 10, 5 μg/mL, followed by serial dilutions) were used as the test system. Cross-reactivity was assessed using unrelated recombinant proteins, including outer membrane proteins (OMP16, OMP19), ribosomal protein (L7/L12), and inner membrane-associated lipoprotein (IalB), as well as bovine serum albumin (BSA). Phosphate-buffered saline (PBS, pH 7.2–7.4) was used as a negative control. Specificity was confirmed by the absence of precipitation rings in control samples and the formation of distinct, symmetrical rings only in the presence of the target antigen.

The square of the precipitation ring diameter (D^2^, mm^2^) was calculated by squaring the measured ring diameter (D, mm). The relative antigen concentration (C/C_0_) was defined as the ratio of the tested recombinant SOD protein concentration (C) to the initial concentration (C_0_). Quantitative evaluation was performed according to the classical Mancini principle, whereby D^2^ is directly proportional to antigen concentration at diffusion equilibrium.

The evaluation was based on ring morphology (clarity and symmetry), reproducibility of measurements, and quantitative parameters (D and D^2^). For each series, measurements were performed in triplicate (*n* = 3) with calculation of D_mean, D^2^_mean, standard deviation (SD), and coefficient of variation (CV, %). Conditions providing distinct and symmetrical ring formation, a linear relationship between D^2^ and antigen concentration and CV ≤ 10% were considered optimal.

#### 2.6.2. Sensitivity

Assay sensitivity was defined as the minimum concentration of SOD1 capable of producing reproducible and visually distinguishable precipitation rings. Twofold serial dilutions of the protein in the concentration range of 10–0.078 μg/mL were analyzed. Each concentration was tested in three independent replicates (*n* = 3). Following incubation, precipitation ring diameters were measured with an accuracy of 0.1 mm. Mean diameter values (D_mean), standard deviation (SD), and coefficient of variation (CV, %) were calculated. The limit of detection (LOD) was defined as the minimum antigen concentration at which reproducible precipitation rings with CV ≤ 10% were observed.

#### 2.6.3. Linearity

Assay linearity was evaluated based on the relationship between the square of the precipitation ring diameter (D^2^) and antigen concentration. Concentrations ranging from 10 to 0.625 μg/mL were analyzed (*n* = 3 for each concentration). The calibration curve was constructed using mean values of squared ring diameters (D^2^_mean). Linearity was assessed by linear regression analysis with calculation of the coefficient of determination (R^2^). An R^2^ value ≥ 0.95 was considered acceptable in accordance with validation requirements for bioanalytical methods [17].

#### 2.6.4. Precision of the Assay

Precision (repeatability) was evaluated at two SOD1 concentration levels (20 and 5 μg/mL). For each concentration, three independent assay series with three replicates each were performed (*n* = 9). The following parameters were calculated: D_mean, mean ring diameter; SD, standard deviation; and CV, coefficient of variation (%), according to the following formula:

CV = SD/D_mean × 100

An assay coefficient of variation not exceeding 10% was considered an acceptable criterion [17].

#### 2.6.5. Robustness of the Assay

Assay robustness was evaluated under controlled variations of experimental conditions, including incubation temperature (25 ± 3 °C), agarose layer thickness (2.5–3.0 mm), incubation time (20–24 h), as well as operator- and reagent lot-related factors. The analysis was performed using SOD1 concentrations of 20 and 5 μg/mL. Each experimental condition was evaluated in triplicate to assess assay repeatability. The assessment was based on the squared diameters of precipitation rings (D^2^). The assay was considered robust when variations in the evaluated experimental conditions had no observable effect on assay performance and the coefficient of variation remained ≤10% [17].

### 2.7. Statistical Analysis

Statistical analysis was performed using GraphPad Prism software (version 10.0, GraphPad Software, San Diego, CA, USA) [25]. Quantitative data obtained from replicate measurements are presented as mean ± standard deviation (SD). The coefficient of variation (CV, %) was calculated to evaluate assay repeatability and precision. Linear regression analysis was performed to assess the relationship between the square of the precipitation ring diameter (D^2^) and the relative antigen concentration (C/C_0_), and the coefficient of determination (R^2^) was used to evaluate the goodness of fit.

## 3. Results

### 3.1. Hyperimmune Sera

The dynamics of hyperimmune serum antibody titers in sheep following hyperimmunization with recombinant SOD protein were evaluated using single radial immunodiffusion (SRID/AGID) and enzyme-linked immunosorbent assay (ELISA).

**Figure 3:**
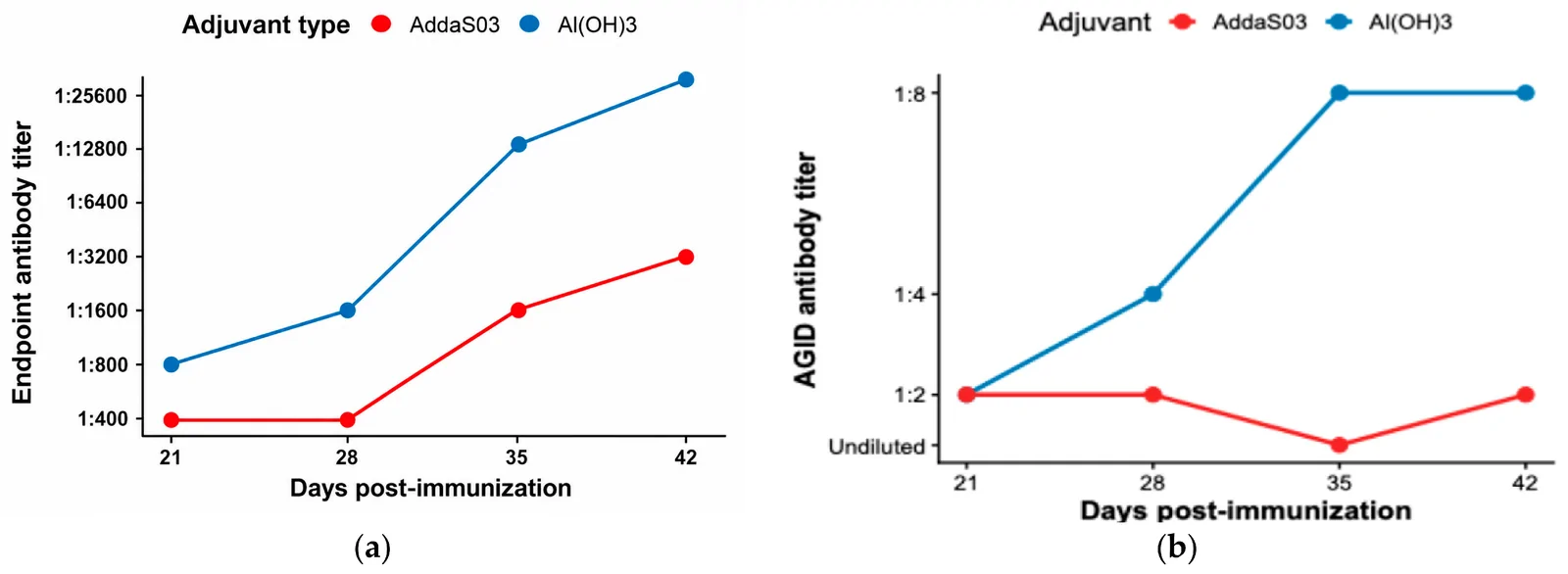
Kinetics of the humoral immune response in sheep following intralymphatic immunization with recombinant SOD protein formulated with different adjuvants. (**a**) Endpoint antibody titers determined by ELISA. (**b**) Antibody titers determined by AGID. Sheep were immunized with recombinant SOD protein formulated with either aluminum hydroxide [Al(OH)_3_] or AddaS03 (1:1, *v*/*v*). Blood samples were collected on Days 21, 28, 35, and 42 after primary immunization to evaluate the kinetics of the humoral immune response. ELISA titers are expressed as endpoint serum dilutions, whereas AGID titers represent the highest serum dilution producing a visible precipitation line.

Antibody levels increased over time in both experimental groups; however, the magnitude of the immune response differed substantially depending on the adjuvant used (Table 1, Figure 3).

In the aluminum hydroxide group, antibody titers determined by ELISA increased progressively from 1:800 on Day 21 to 1:25,600 by Day 42. A similar trend was observed using the AGID assay, where antibody titers increased from 1:2 to 1:8.

In the AddaS03 adjuvant group, antibody levels were substantially lower: ELISA titers increased from 1:400 to 1:3200, whereas AGID analysis demonstrated a weak and inconsistent antibody response, with maximum titers reaching only 1:2.

Although no statistically significant differences were observed between the groups (*p* = 0.10), the consistent trend indicated the superiority of aluminum hydroxide in promoting the formation of high-affinity antibodies. The obtained hyperimmune sera were characterized by high titers and specificity and considered suitable for use as an antibody source in the SRID assay.

### 3.2. Optimization of the Single Radial Immunodiffusion Assay

**Figure 4:**
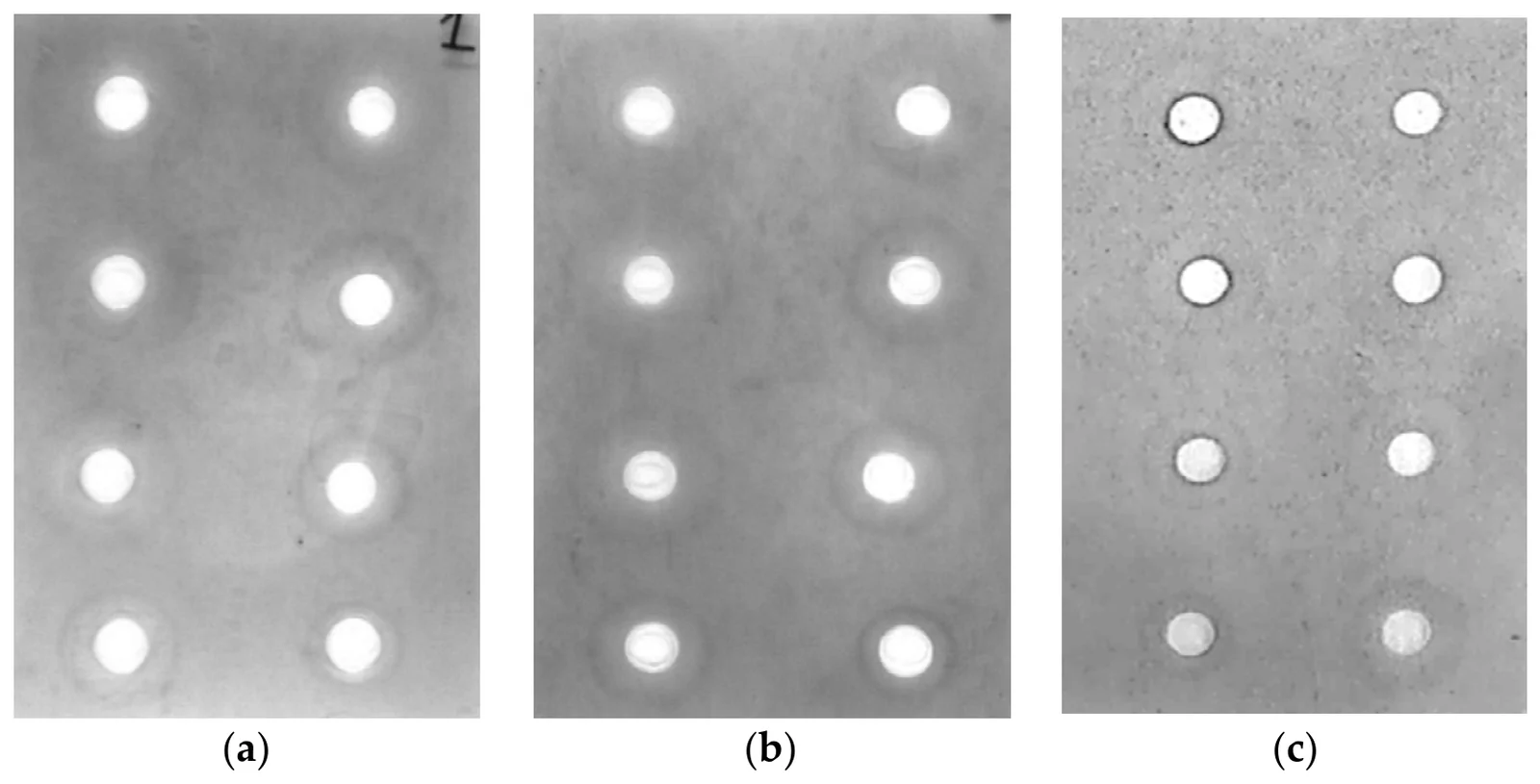
Formation of precipitation rings in the single radial immunodiffusion (SRID) assay using three independent batches of hyperimmune sera against recombinant SOD protein. (**a**) Hyperimmune serum batch No. 1; (**b**) hyperimmune serum batch No. 2; (**c**) hyperimmune serum batch No. 3. In each panel, recombinant SOD protein was tested at initial concentrations of 20 and 10 μg/mL and at serial relative concentrations obtained by dilution (1.00, 0.75, 0.50, and 0.25, corresponding to 1:0, 3:1, 1:1, and 1:3, respectively). The resulting precipitation ring diameters are summarized in Table 2.

To confirm the suitability of the optimized assay conditions, the SRID assay was evaluated using three independent batches of hyperimmune sera against recombinant SOD protein and recombinant SOD protein at concentrations of 20 and 10 μg/mL (Figure 4, Table 2).

Figure 4a–c shows the formation of precipitation rings following the interaction of SOD antigen with antisera from different batches. In all cases, distinct and reproducible precipitation ring formation was observed. As the antigen concentration decreased, a corresponding reduction in ring diameter was detected, consistent with the theoretical principles of the SRID assay, according to which the square of the precipitation ring diameter (D^2^) is proportional to antigen concentration.

Antisera from all batches demonstrated comparable precipitation ring size and clarity, indicating the stability of their immunoreactive properties and the reproducibility of the obtained results.

Quantitative analysis (Table 2) demonstrated that at an antigen concentration of 20 μg/mL, precipitation ring diameters for batch No. 1 ranged from 18.0 to 12.1 mm, for batch No. 2 from 17.2 to 11.6 mm and for batch No. 3 from 17.1 to 10.9 mm. At an antigen concentration of 10 μg/mL, the corresponding values were 15.1–10.3 mm, 15.2–9.5 mm and 15.0–9.4 mm, respectively.

Differences between antisera batches did not exceed 5–8% at most experimental points, showing high reproducibility of the immunodiffusion reaction and comparable activity among different antibody batches.

**Figure 5:**
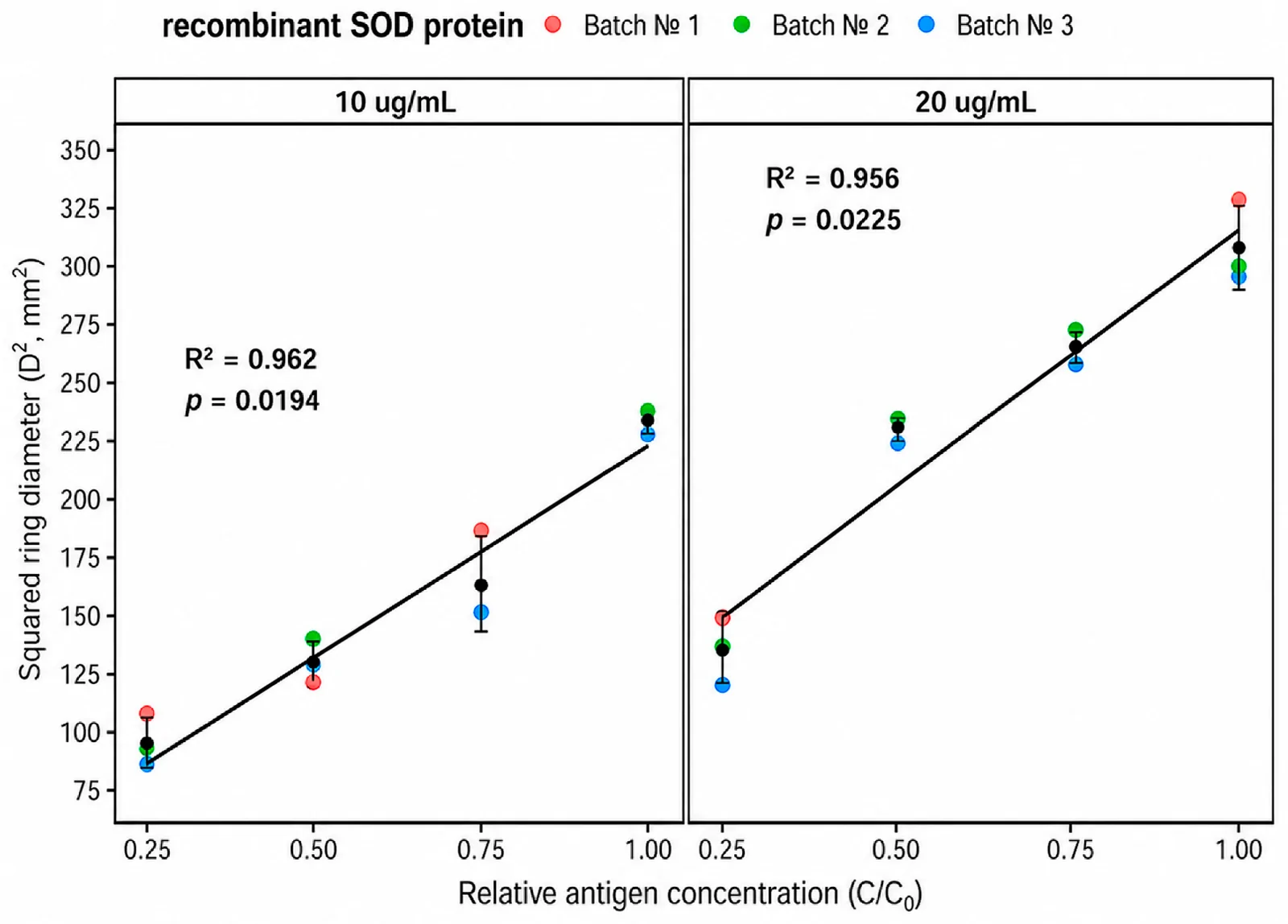
Linear relationship between recombinant SOD protein concentration and the square of the precipitation ring diameter (D^2^) in the single radial immunodiffusion (SRID) assay. Dependence of the square of the precipitation ring diameter (D^2^) on the relative antigen concentration (C/C_0_) in the single radial immunodiffusion (SRID) assay at antigen concentrations of 10 and 20 μg/mL. Individual values for the three recombinant SOD protein batches are shown as colored dots, whereas black markers represent mean values ± standard deviation (SD). Data are presented as mean ± SD (*n* = 3). Linear regression analysis confirmed a strong linear relationship (*R*^2^ = 0.956–0.962).

A decrease in antigen concentration was accompanied by a gradual reduction in precipitation ring diameter. The relationship between the square of the ring diameter (D^2^) and antigen concentration exhibited a linear pattern, consistent with the theoretical principles of the SRID assay (Figure 5).

The square of the precipitation ring diameter (D^2^) demonstrated a linear relationship with the relative antigen concentration (C/C_0_), consistent with the theoretical principles underlying the single radial immunodiffusion (SRID) assay.

Linear regression analysis of the averaged data demonstrated a clear concentration-dependent response at both antigen levels (10 and 20 μg/mL), indicating the suitability of the assay for quantitative antigen determination.

The obtained results demonstrated that the optimized assay conditions provided stable and reproducible precipitation ring formation regardless of the antiserum batch, confirming the robustness and reliability of the SRID assay.

### 3.3. Specificity of the SRID Assay

**Figure 6:**
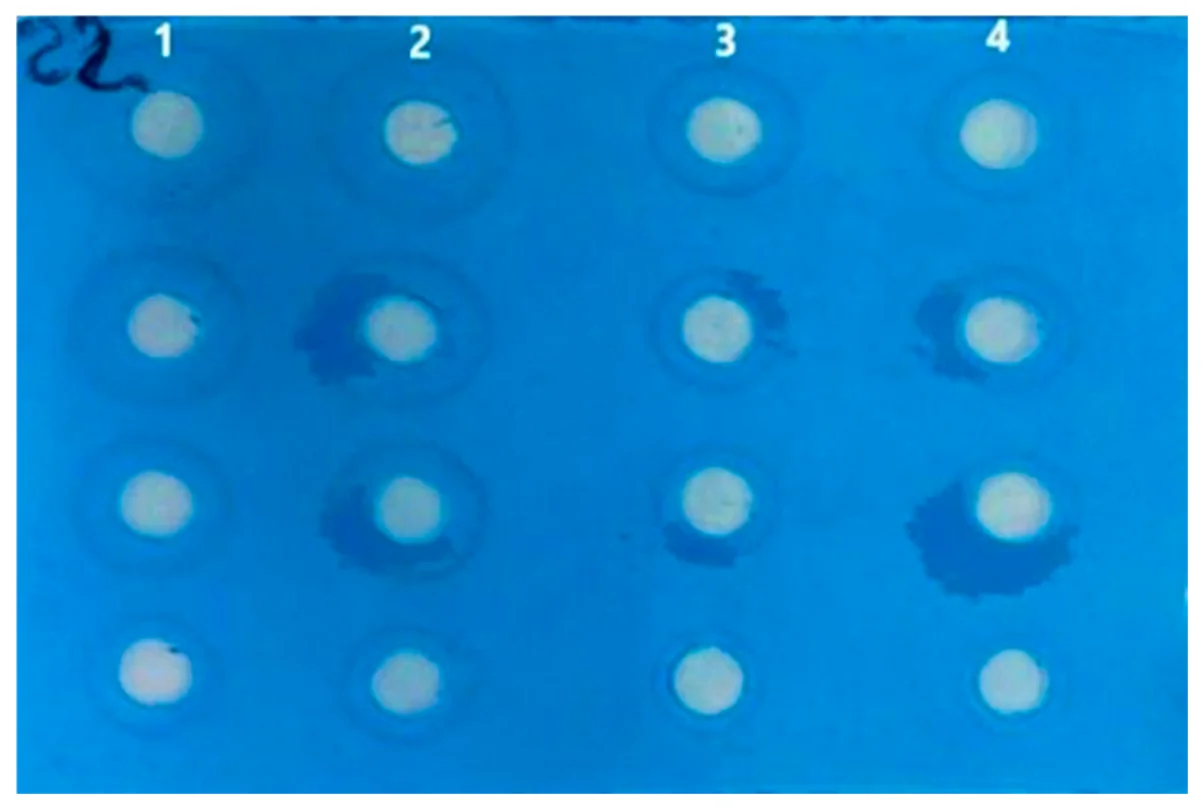
Precipitation rings formed by the recombinant human SOD1 reference standard and recombinant SOD protein batch No. 1 in the single radial immunodiffusion (SRID) assay: 1—recombinant human SOD1 reference standard, 20 μg/mL; 2—recombinant SOD protein batch No. 1, 20 μg/mL; 3—recombinant human SOD1 reference standard, 10 μg/mL; 4—recombinant SOD protein batch No. 1, 10 μg/mL. The figure illustrates the comparative precipitation ring formation obtained with the commercial SOD1 reference standard and the recombinant SOD protein at two antigen concentrations (20 and 10 μg/mL).

At concentrations of 20 and 10 μg/mL, both the recombinant human SOD1 reference standard and recombinant SOD protein batch No. 1 formed distinct precipitation rings (Figure 6). The mean precipitation ring diameters for recombinant SOD protein batch No. 1 were 11.13 mm at 20 μg/mL and 8.63 mm at 10 μg/mL (Table 3). The deviation from the recombinant human SOD1 reference standard did not exceed 4.35%, which was within the predefined acceptance criterion (≤10%).

The precipitation ring diameters obtained for recombinant SOD protein batch No. 1 were comparable with those obtained for the recombinant human SOD1 reference standard at both concentration levels (Table 3).

**Figure 7:**
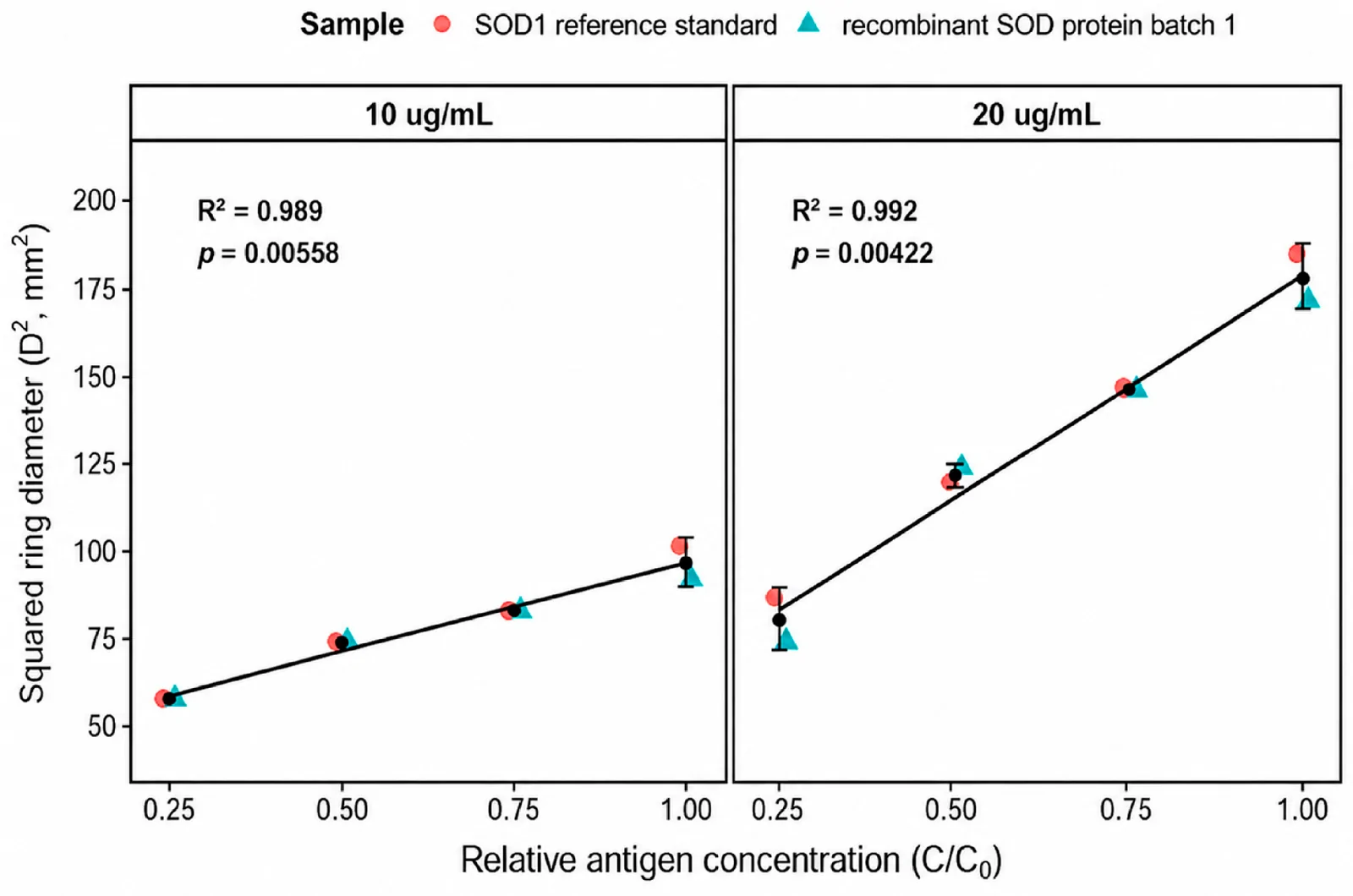
Linear relationship between D^2^ and relative antigen concentration (C/C_0_) in the SRID assay. High coefficients of determination (R^2^ > 0.98) confirmed the linearity of the assay and the comparability of the reference standard and recombinant protein.

To confirm the quantitative comparability of the samples, the relationship between the square of the precipitation ring diameter (D^2^) and the relative antigen concentration was analyzed (Figure 7).

In contrast, the negative control, BSA, as well as the recombinant proteins OMP16, L7/L12, IalB, and OMP19, did not form precipitation rings, indicating the analytical specificity of the assay and the absence of cross-reactivity with unrelated proteins. These findings are consistent with the quantitative analysis results (Table 4), further confirming the reproducibility of the assay.

As shown in Table 4, the SOD1 (reference standard) and recombinant SOD protein batch No. 1 formed precipitation rings of comparable diameter at concentrations of 20 and 10 μg/mL. The mean ring diameters (D_mean) and corresponding D^2^ values obtained for the test sample were close to those of the reference standard.

Additionally, the relationship between the square of the precipitation ring diameter (D^2^) and the relative antigen concentration was analyzed (Figure 7). Both the reference standard and recombinant protein demonstrated a linear relationship with high coefficients of determination (R^2^ > 0.98). The parallelism of the calibration curves indicates a similar antigen–antibody interaction pattern and comparable antigenic activity of the samples.

The obtained results confirm the high analytical specificity of the SRID assay and the selectivity of antibodies against SOD protein, while also demonstrating the equivalence of the antigenic properties of recombinant SOD protein batch No. 1 compared with the SOD1 (reference standard).

### 3.4. Sensitivity of the SRID Assay

Assay sensitivity was evaluated using serial dilutions of recombinant SOD protein batch No. 1 in the concentration range of 10–0.078 μg/mL. Within the concentration range of 10–0.625 μg/mL, distinct and clearly visible precipitation rings with reproducible diameter values were observed. At a concentration of 0.312 μg/mL, a measurable precipitation ring was still observed; however, its boundaries were less distinct than those obtained at higher antigen concentrations, allowing this concentration to be considered the threshold level of detection. The mean ring diameter was 2.90 ± 0.10 mm with a coefficient of variation of CV = 3.4%, indicating acceptable reproducibility at the lower limit of detection.

**Figure 8:**
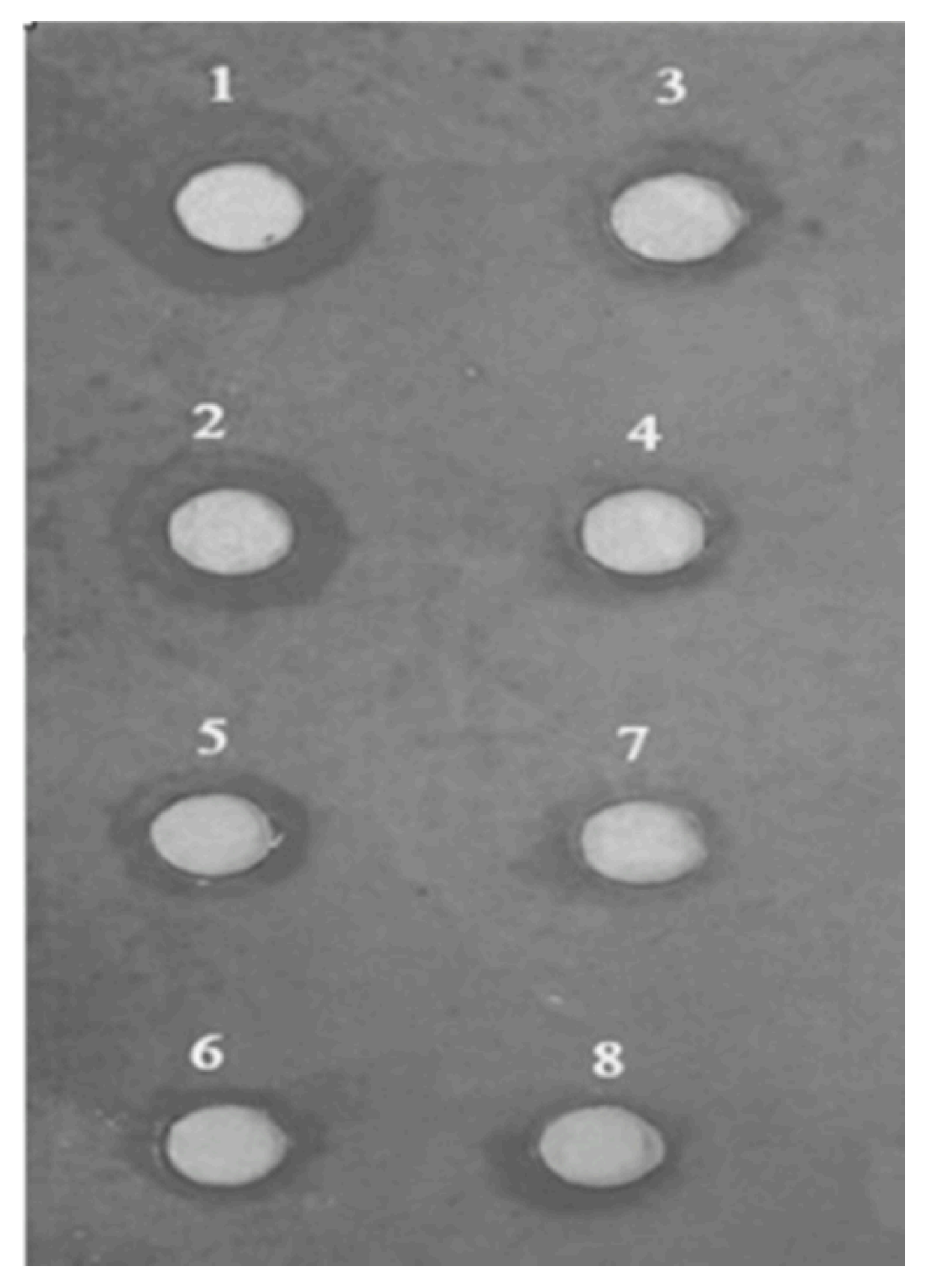
Precipitation rings obtained at serial dilutions of recombinant SOD protein in the single radial immunodiffusion (SRID) assay: 1—10 μg/mL; 2—5 μg/mL; 3—2.5 μg/mL; 4—1.25 μg/mL; 5—0.625 μg/mL; 6—0.312 μg/mL (limit of detection, LOD); 7—0.156 μg/mL; 8—0.078 μg/mL. Distinct precipitation rings were observed down to 0.625 μg/mL, whereas a weak but measurable ring was detected at 0.312 μg/mL, corresponding to the assay limit of detection.

At further dilutions (0.156 and 0.078 μg/mL), distinct precipitation rings were no longer observed, and the weak diffusion zones detected did not meet the criteria for a positive signal (Figure 8).

Quantitative results of precipitation ring diameter measurements are presented in Table 5.

The limit of detection (LOD) for recombinant SOD protein determined by the SRID assay was 0.312 μg/mL. The obtained data confirm that the assay possesses sufficient sensitivity for monitoring protein content in the vaccine candidate formulation.

### 3.5. Linearity

**Figure 9:**
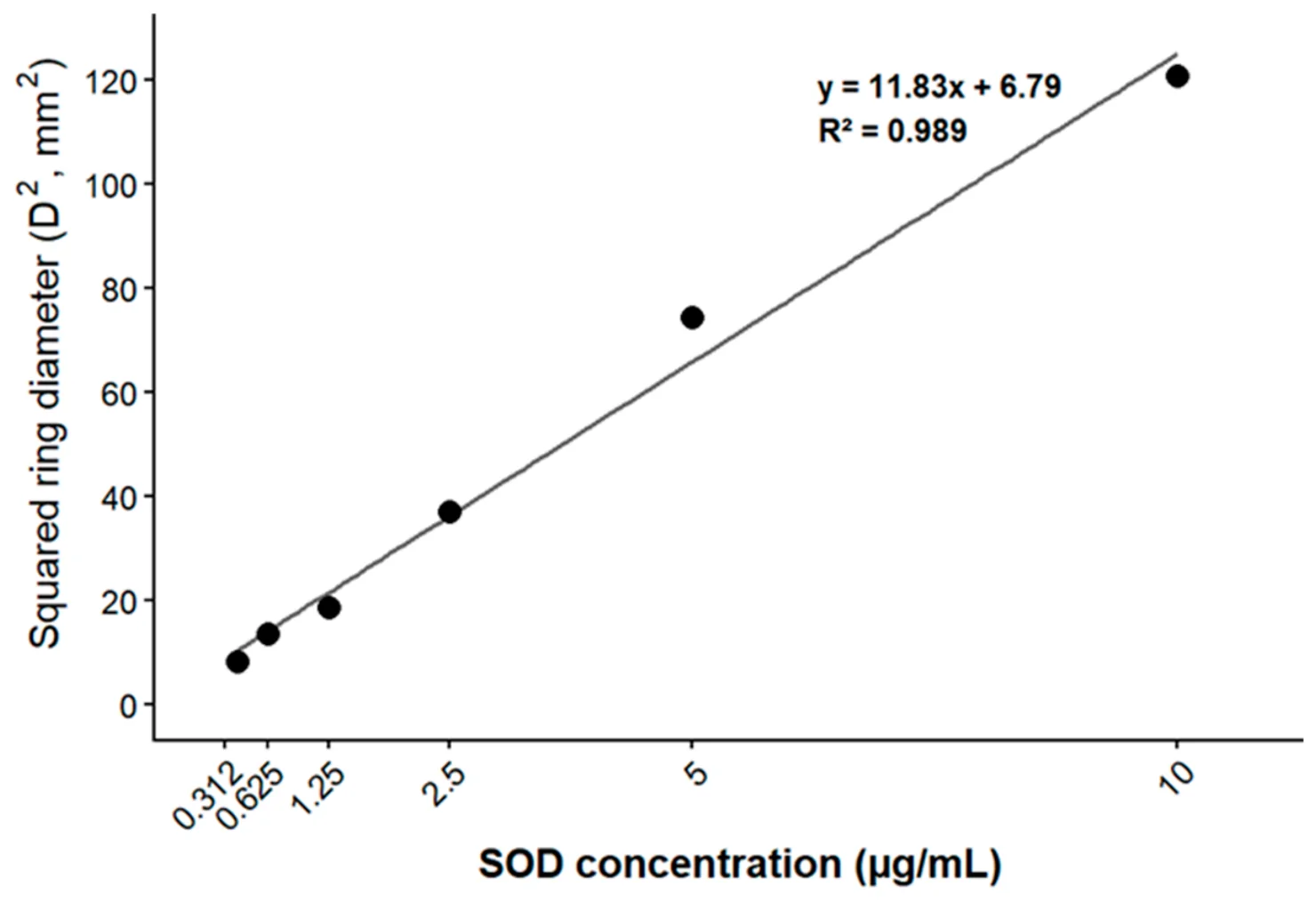
Calibration curve for quantitative determination of recombinant SOD protein by the single radial immunodiffusion (SRID) assay. Data are presented as mean ± standard deviation (SD) (*n* = 3). Linear regression analysis demonstrated a strong linear relationship between the square of the precipitation ring diameter (D^2^) and recombinant SOD protein concentration (R^2^ = 0.989).

Analysis of the relationship between the square of the precipitation ring diameter (D^2^) and recombinant SOD protein concentration demonstrated a linear correlation within the concentration range of 10–0.625 μg/mL. The constructed calibration curve exhibited a pronounced linear pattern, as confirmed by the high coefficient of determination value (R^2^ = 0.989) (Figure 9).

The obtained data (Figure 9) demonstrated a high degree of correlation between antigen concentration and precipitation ring diameter within the studied concentration range. At a concentration of 0.312 μg/mL, small but reproducible precipitation rings were observed; however, this data point was excluded from the linear regression analysis because it corresponded to the threshold region of assay sensitivity.

The obtained results demonstrated that the SRID assay provides a linear relationship between analytical signal and recombinant SOD protein concentration within the range of 10–0.625 μg/mL and is suitable for quantitative antigen determination within this concentration interval.

### 3.6. Precision

The results of the assessment of intra-assay precision (repeatability) of the SRID assay are presented in Table 6.

For the 20 μg/mL concentration, the mean precipitation ring diameter was 13.07 mm (*n* = 9) with a standard deviation of 0.16 mm. The coefficient of variation was 1.28%. For the 5 μg/mL concentration, the mean ring diameter was 8.66 mm (*n* = 9) with a standard deviation of 0.10 mm. The coefficient of variation was 1.21%. The obtained coefficient of variation values were below the established acceptance criterion (≤10%), indicating high repeatability of the assay. The low variability of measurements indicates stable precipitation ring formation and reproducible antigen–antibody interactions within the agarose matrix.

The obtained data demonstrate high intra-assay precision of the SRID assay within the studied concentration range and confirm its suitability for the quantitative determination of recombinant SOD protein.

### 3.7. Robustness

The analysis demonstrated that variations in incubation temperature, agarose layer thickness, incubation time, operator, and reagent lot had no observable effect on assay performance under the conditions evaluated.

**Figure 10:**
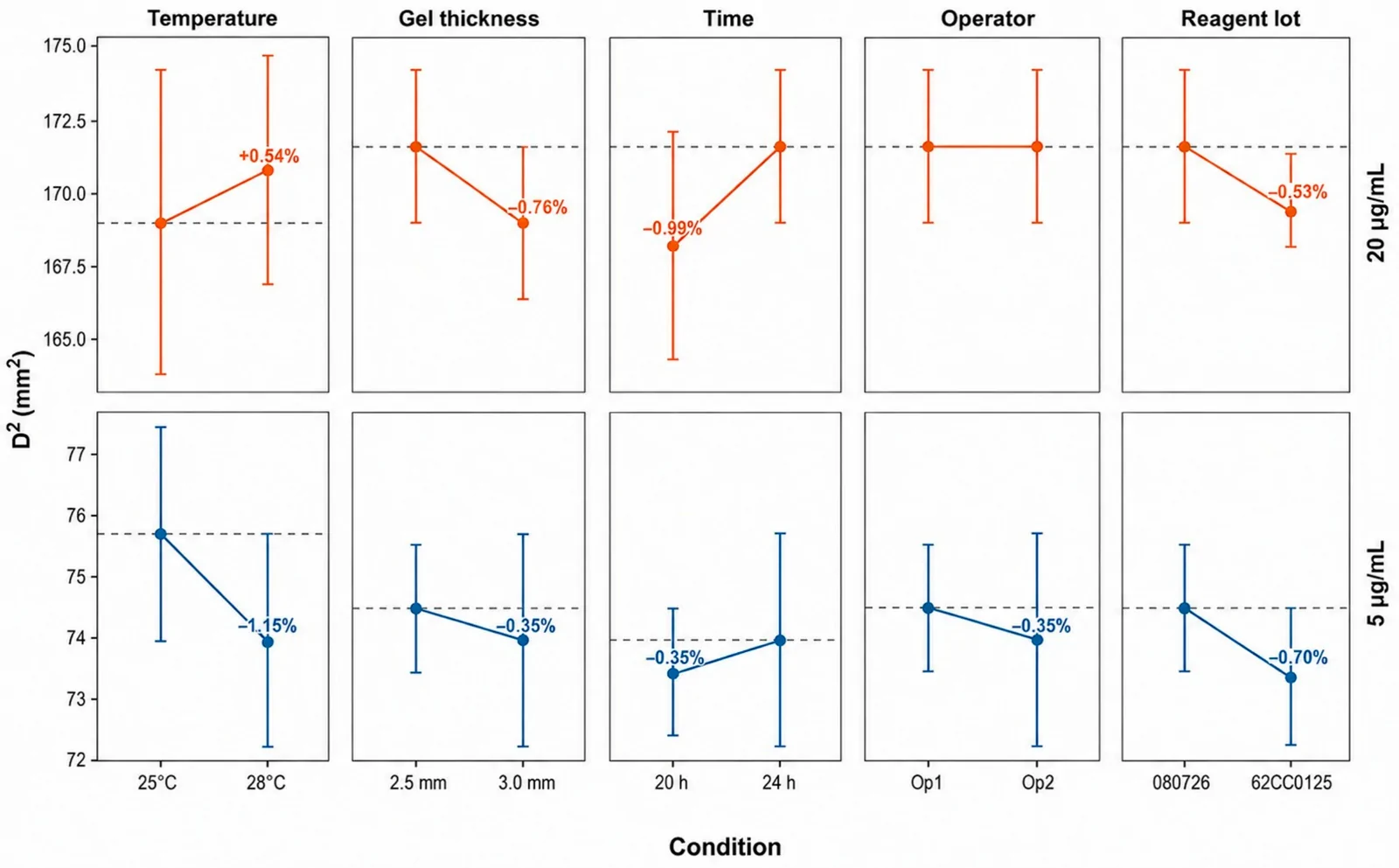
Effect of variations in analytical conditions on the square of the precipitation ring diameter (D^2^) in the single radial immunodiffusion (SRID) assay. Data are presented as mean ± standard deviation (SD) (*n* = 3). Deviations from the standard conditions did not exceed ±1% under the conditions evaluated. Orange symbols represent the results obtained for the 20 μg/mL SOD concentration, whereas blue symbols represent the results obtained for the 5 μg/mL SOD concentration. The dashed horizontal lines indicate the reference D^2^ values obtained under the standard analytical conditions. Deviations from the standard conditions did not exceed ±1% under the evaluated conditions.

For the 20 μg/mL concentration, mean precipitation ring diameters ranged from 12.97 to 13.10 mm, with coefficients of variation ranging from 0.46% to 1.54%. For the 5 μg/mL concentration, the corresponding values ranged from 8.57 to 8.70 mm and from 0.70% to 1.16%, respectively. Deviations of the mean values relative to the standard conditions did not exceed ±1%, indicating that the investigated factors had a negligible effect on the analytical results (Table 7, Figure 10).

The obtained values of mean precipitation ring diameter (D_mean), standard deviation (SD), coefficient of variation (CV), and relative deviation (Δ, %) are presented in Table 7.

The obtained data indicate that the assay is resistant to minor variations in analytical conditions and confirm its robustness.

Thus, the developed SRID assay is characterized by high tolerance to changes in analytical parameters and can be applied in routine laboratory practice without a significant risk of result distortion.

## 4. Discussion

The present study demonstrated that hyperimmunization of sheep with recombinant SOD protein induces a humoral immune response, the magnitude and kinetics of which are substantially influenced by the adjuvant used. The obtained data showed that aluminum hydroxide produced higher antibody titers compared with the emulsion-based adjuvant AddaS03, as confirmed by both ELISA and agar gel immunodiffusion (AGID) assays.

The superiority of aluminum hydroxide is consistent with current understanding of the mechanisms of action of aluminum-containing adjuvants. These compounds are known to promote antigen depot formation at the injection site, enhance antigen uptake by antigen-presenting cells, and activate innate immune pathways, including inflammasome-associated signaling, ultimately leading to enhanced B-cell activation and antibody production [9,26]. In contrast, squalene-based emulsion adjuvants (such as AddaS03/AS03) are known to induce broader immune responses involving cellular immune mechanisms; however, their effects on humoral immunity may vary depending on antigen characteristics and immunization conditions [27]. This may explain the lower antibody titers observed in the present study.

Although statistically significant differences between the groups were not observed, the consistent trend toward higher antibody titers in the aluminum hydroxide group may still be considered biologically relevant. Similar findings have been reported in immunological studies involving limited numbers of experimental animals, where interindividual variability may reduce the statistical power of the analysis [8].

The optimized single radial immunodiffusion (SRID) assay demonstrated high analytical reliability. The observed linear relationship between the square of the precipitation ring diameter (D^2^) and antigen concentration is consistent with the classical Mancini model and confirms the suitability of the assay for quantitative protein determination. The high coefficients of determination (R^2^ > 0.95) further demonstrate the accuracy and reliability of the developed method and agree with previous studies validating immunodiffusion-based assays under standardized analytical conditions [17].

The developed SRID assay demonstrated a limit of detection (LOD) of 0.312 μg/mL, providing sufficient analytical sensitivity for quantitative determination of recombinant SOD protein within the validated concentration range.

Importantly, direct comparison of absolute detection limits among different analytical methods should be interpreted with caution, as the apparent analytical sensitivity depends not only on the analytical platform itself but also on antigen format, calibration strategy, sample matrix, and the immunochemical properties of the capture or precipitating antibodies. In SRID, quantitative determination is inherently based on antigen diffusion and precipitation within an antibody-containing agarose gel and is therefore influenced by the specificity of the precipitating antiserum, as originally described in classical immunodiffusion studies.

In recent years, several analytical platforms have been developed for the detection and quantification of recombinant proteins, including recombinant His_6_-tagged proteins. These include aggregation-induced emission (AIE)-based competitive immunoassays [28], gold nanoparticle (AuNP)-based colorimetric immunoassays [29,30], label-free electrochemical sensing platforms [31], and competitive electrochemical immunosensors [32].

Among these approaches, competitive electrochemical immunosensors have been specifically designed for the detection of recombinant His_6_-tagged proteins by exploiting the coordination interaction between the hexahistidine tag and functionalized nanomaterials or metal–organic frameworks. This strategy provides excellent analytical sensitivity but requires modified electrodes and specialized instrumentation. For example, Chang et al. [32] developed a competitive electrochemical immunosensor for the quantification of recombinant His_6_-tagged SARS-CoV-2 nucleocapsid protein, achieving a linear detection range of 1 pg/mL to 1 ng/mL. Similarly, Gao et al. [28] reported an AIE-based competitive “signal-on” immunoassay using recombinant His_6_-tagged procalcitonin as a model analyte, with a linear range of 0.01–10 pg/mL and a limit of detection of 8 pg/mL.

Gold nanoparticle-based colorimetric immunoassays have also been widely applied for recombinant protein detection. Liu et al. [30] developed a highly sensitive colorimetric immunoassay based on scFv-functionalized gold nanoparticles, achieving a detection limit of 1.7 nM. The high analytical performance was attributed to the small size of the recombinant scFv antibody fragment and its covalent conjugation to gold nanoparticles. Nevertheless, despite their rapid visual readout and high sensitivity, AuNP-based assays remain susceptible to variations in nanoparticle stability, environmental conditions, and the reproducibility of nanoparticle functionalization [29,30,33].

Label-free electrochemical sensing platforms represent another promising approach for recombinant protein analysis. Ratautaite et al. [34] developed a molecularly imprinted polypyrrole (MIP-Ppy) electrochemical sensor for the detection of recombinant SARS-CoV-2 spike glycoprotein, demonstrating a higher analytical response than the corresponding non-imprinted sensor. However, although electrochemical biosensors provide excellent sensitivity and rapid analysis, they generally require specialized instrumentation and careful optimization of sensor fabrication [31,34].

In contrast to these advanced analytical platforms, the Mancini single radial immunodiffusion (SRID) assay is based on the direct interaction between antigen and specific antibodies within an agarose gel and enables direct immunochemical quantification of recombinant protein without the need for enzymatic, fluorescent, or nanomaterial-based labels. Although SRID requires longer incubation times and exhibits lower analytical sensitivity than modern biosensor-based approaches, it offers excellent reproducibility, methodological simplicity, low implementation cost, and good inter-laboratory comparability. These characteristics make SRID particularly suitable for the standardization and routine quality control of recombinant SOD protein in the developed brucellosis vaccine candidate [24,35,36].

The absence of cross-reactivity with unrelated proteins (OMP16, L7/L12, IalB, and OMP19) and bovine serum albumin (BSA) indicates high analytical specificity of the assay and strong selectivity of the obtained antisera. This observation is consistent with current concepts suggesting that the specificity of immunodiffusion-based methods primarily depends on antibody quality and the uniqueness of antigenic epitopes [22].

Precision analysis demonstrated low coefficients of variation (CV < 2%), indicating high assay repeatability. The observed CV values (<2%) were substantially lower than the acceptance limits commonly applied for quantitative analytical methods, demonstrating excellent repeatability of the developed SRID assay [13,22]. Therefore, the developed assay demonstrated excellent repeatability and exceeded the generally accepted precision criteria for quantitative analytical methods.

Robustness analysis demonstrated that variations in incubation temperature, agarose layer thickness, reaction time, operator, and reagent batch did not significantly affect assay performance under the evaluated conditions. These findings confirm the stability of the method under varying analytical conditions and support its suitability for routine laboratory application.

In summary, the obtained results confirm that the SRID assay represents a validated, reproducible and specific analytical tool for the quantitative determination of recombinant SOD protein. Also, antisera generated using aluminum hydroxide represent a reliable source of high-affinity antibodies and may be effectively applied in immunodiffusion assays and vaccine antigen standardization procedures.

## 5. Conclusions

Hyperimmunization of sheep with recombinant SOD protein induced a pronounced humoral immune response, with aluminum hydroxide demonstrating higher immunostimulatory activity compared with AddaS03. The developed SRID assay exhibited high specificity, reproducibility, precision, and robustness. A linear relationship between antigen concentration and precipitation ring diameter was observed within the concentration range of 10–0.625 μg/mL, while the limit of detection was 0.312 μg/mL. No cross-reactivity with unrelated proteins was detected, and all validation parameters met the established acceptance criteria. Overall, the developed SRID assay represents a validated and reliable tool for quantitative determination of recombinant SOD protein and may be applied in quality control of brucellosis vaccine formulations.

## Figures and Tables

**Table 1:** Dynamics of antibody titers in sheep immunized with recombinant SOD protein formulated with aluminum hydroxide or AddaS03, as determined by agar gel immunodiffusion (AGID) and enzyme-linked immunosorbent assay (ELISA).

Animals (Group)	Antigen	Adjuvant	Results							
			Day 21		Day 28		Day 35		Day 42	
			AGID	ELISA	AGID	ELISA	AGID	ELISA	AGID	ELISA
Sheep Group #1	Recombinant SOD protein	Aluminum hydroxide (0.2%, final concentration)	1:2	1:800	1:4	1:1600	1:8	1:12,800	1:8	1:25,600
Sheep Group #2		AddaS03 (1:1, *v/v* with antigen)	1:2	1:400	1:2	1:400	undiluted serum	1:1600	1:2	1:3200

Note: Antibody titers are expressed as the reciprocal of the highest serum dilution yielding a positive result.

**Table 2:** Reproducibility of the single radial immunodiffusion (SRID) assay for recombinant SOD protein using three independent batches of hyperimmune sera.

Relative Concentration (C/C_0_)	Batch No. 1		Batch No. 2		Batch No. 3	
Dilution	20	10	20	10	20	10
	Ring Diameter, mm					
1 (1:0)	18	15.1	17.2	15.2	17.1	15
0.75 (3:1)	16.1	13.5	16.3	12.2	16	12.2
0.50 (1:1)	15	11	15	11.7	14.8	11.3
0.25 (1:3)	12.1	10.3	11.6	9.5	10.9	9.4

**Table 3:** Measurement results of precipitation ring diameters for the SOD1 reference standard and recombinant SOD protein batch No. 1 determined by the SRID assay.

Serum Dilution in Agar	SOD1 (Reference Standard)		Recombinant SOD Protein, Batch No. 1	
	20 μg/mL	10 μg/mL	20 μg/mL	10 μg/mL
	Ring Diameter, mm			
1 (1:0)	13.5	10.0	13.0	9.5
0.75 (3:1)	12.0	9.0	12.0	9.0
0.50 (1:1)	10.8	8.5	11.0	8.5
0.25 (1:3)	9.2	7.5	8.5	7.5

**Table 4:** Precipitation ring diameters for recombinant SOD protein and heterologous control proteins in the SRID assay.

Concentration (μg/mL)	Sample	1 (1:0)	0.75 (3:1)	0.50 (1:1)	0.25 (1:3)
20	PO-SOD-1	13.5	12.0	10.8	9.2
20	SOD batch No.1	13.0	12.0	11.0	8.5
20	NC (negative control)	ND	ND	ND	ND
20	OMP16	ND	ND	ND	ND
20	L7/L12	ND	ND	ND	ND
20	IalB	ND	ND	ND	ND
20	OMP19	ND	ND	ND	ND
20	BSA	ND	ND	ND	ND
10	SOD1 (reference standard)	10.0	9.0	8.5	7.5
10	SOD batch No.1	9.5	9.0	8.5	7.5
10	NC (negative control)	ND	ND	ND	ND
10	OMP16	ND	ND	ND	ND
10	L7/L12	ND	ND	ND	ND
10	IalB	ND	ND	ND	ND
10	OMP19	ND	ND	ND	ND
10	BSA	ND	ND	ND	ND

**Note:** ND, not detected (no precipitation rings observed).

**Table 5:** Diameters of precipitation rings at different concentrations of recombinant SOD protein batch No. 1.

Concentration, μg/mL	Replicate 1 (mm)	Replicate 2 (mm)	Replicate 3 (mm)	Dmean (mm)	CV (%)	Evaluation
10	11.0	10.8	11.2	11.00	1.8	+
5	8.8	8.5	8.6	8.63	1.7	+
2.5	6.1	5.9	6.3	6.10	3.3	+
1.25	4.5	4.2	4.3	4.33	3.9	+
0.625	3.8	3.6	3.7	3.70	2.7	+
0.312	3.0	2.9	2.8	2.90	3.4	“±” threshold-level signal.
0.156	2.0	2.1	2.0	2.03	2.5	–
0.078	1.5	1.4	1.5	1.47	3.0	–

**Note:** “+”—distinct precipitation ring; “±”—weak but measurable and reproducible ring corresponding to the threshold level; “−”—absence of a distinct analytical signal; “CV”—coefficient of variation (%).

**Table 6:** Precision of the single radial immunodiffusion (SRID) assay for quantitative determination of recombinant SOD protein.

Concentration, μg/mL	Replicate 1 ± SD (mm)	Replicate 2 ± SD (mm)	Replicate 3 ± SD (mm)	D_mean (mm)	CV (%)	n
20	13.0 ± 0.20 (mm)	13.1 ± 0.20 (mm)	13.1 ± 0.10 (mm)	13.07	1.28	9
5	8.7 ± 0.10 (mm)	8.6 ± 0.10 (mm)	8.67 ± 0.12 (mm)	8.66	1.21	9

Note: D_mean (mm), mean value of all nine measurements (three replicates); SD (mm), standard deviation; CV (%), coefficient of variation calculated as SD/D_mean × 100.

**Table 7:** Robustness of the single radial immunodiffusion (SRID) assay under varying analytical conditions.

Variation Parameter	Condition	Conc., μg/mL	D_Mean ± SD (mm)	CV (%)	Δ (%)	n
Incubation temperature	Standard (25 °C)	20	13.00 ± 0.20	1.54	0	3
	Modified (28 °C)	20	13.07 ± 0.15	1.15	0.54	3
	Standard (25 °C)	5	8.70 ± 0.10	1.15	0	3
	Modified (28 °C)	5	8.60 ± 0.10	1.16	−1.15	3
Gel layer thickness	Standard (2.5 mm)	20	13.10 ± 0.10	0.76	0.77	3
	Modified (3 mm)	20	13.00 ± 0.10	0.77	0	3
	Standard (2.5 mm)	5	8.63 ± 0.06	0.70	−0.77	3
	Modified (3 mm)	5	8.60 ± 0.10	1.16	−0.39	3
Incubation time	Standard (24 h)	20	13.10 ± 0.10	0.76	0.77	3
	Modified (20 h)	20	12.97 ± 0.15	1.16	−1.02	3
	Standard (24 h)	5	8.60 ± 0.10	1.16	0	3
	Modified (20 h)	5	8.57 ± 0.06	0.70	−0.39	3
Operators	Researcher 1	20	13.10 ± 0.10	0.76	0.77	3
	Researcher 2	20	13.10 ± 0.10	0.76	0	3
	Researcher 1	5	8.63 ± 0.06	0.70	0	3
	Researcher 2	5	8.60 ± 0.10	1.16	−0.39	3
Reagent lot	Lot: 080726	20	13.10 ± 0.10	0.76	0	3
	Lot: 62CC0125	20	13.03 ± 0.06	0.46	−0.51	3
	Lot: 080726	5	8.63 ± 0.06	0.70	0	3
	Lot: 62CC0125	5	8.57 ± 0.06	0.70	−0.77	3

## Data Availability

The data presented in this study are available from the corresponding author upon reasonable request.

## Abbreviations

The following abbreviations are used in this manuscript:

AGID	Agar Gel Immunodiffusion
Al(OH)_3_	Aluminum Hydroxide
BSA	Bovine Serum Albumin
CV	Coefficient of Variation
D	Precipitation Ring Diameter
D^2^	Square of the Precipitation Ring Diameter
ELISA	Enzyme-Linked Immunosorbent Assay
LOD	Limit of Detection
PBS	Phosphate-Buffered Saline
R^2^	Coefficient of Determination
SD	Standard Deviation
SRID	Single Radial Immunodiffusion
